# Supplementary material for: Are carbon emissions trading and green financial instruments synergistic? -Comprehensive quantitative research based on content analysis
Source: PLoS One. 2024 Mar 7;19(3):e0298601. doi: 10.1371/journal.pone.0298601 (PMC10919601; doi:10.1371/journal.pone.0298601)
Supplement: S1 Data — (ZIP) [file pone.0298601.s001.zip › Data/地级市Z_F工作报告.zip!/╣π╬≈/╬α╓▌╩╨/└┤╫╘╣1⁄2╓┌║┼í╛┬φ┐╦╩2╛▌═°í┐_2003.docx]

各位代表：

现在，我代表市第十一届人民政府向大会作工作报告，请予审议，并请市政协各位委员和其他同志提出意见。

一、2002年工作的回顾

过去的一年，市人民政府在中共梧州市委的领导下，认真贯彻落实江泽民同志"5·31"重要讲话和党的十六大精神，以邓小平理论和"三个代表"重要思想为指导，按照自治区第八次党代会的要求，围绕市委"建设东大门，实现新跨越"的战略部署，抓住我国西部大开发和加入世贸组织的机遇，依靠全市人民，与时俱进，开拓进取，扎实工作，战胜了五次严重的洪涝灾害等各种困难，较好地完成了各项目标任务，全市国民经济稳步发展，各项事业取得新的成绩。

全年主要预期目标全部实现，国民经济发展态势良好。初步统计，全市完成国内生产总值152.25亿元，比上年增长10.5%，创近几年最好水平。其中第一产业完成46.41亿元，增长7.0%；第二产业完成45.58亿元，增长11.5%；第三产业完成60.26亿元，增长12.6%。财政一般预算收入完成12.59亿元，增长11.1%。全社会固定资产投资完成30.13亿元，增长14.5%。全市完成工业增加值38.75亿元，增长10.5%。全市实现外贸出口总额1.78亿美元，增长24.6%，其中市属外贸出口1.16亿美元，增长40.7%。实际利用外资6283万美元，增长2.4%。城镇居民人均可支配收入6282元，增长7.6%。农民人均纯收入1900元，增长6.5%。社会消费品零售总额完成69.5亿元，增长9.8%。人口自然增长率为7.65‰。上述指标完成比预期的好，比前几年好，大部分指标超过全区的平均增长水平。

农业结构调整取得新进展，现代农业实验区和农田水利建设成效明显。农业结构继续优化。全市调减粮食种植面积11.45万亩，其中水稻9.43万亩，全市优质稻比例达70%。新增高效经济作物面积14.8万亩，粮食和经济作物种植面积由上年的1∶0.64调整为1∶0.70。优质和特色农产品生产规模进一步扩大，全市新增桑园面积2.72万亩，名优水果4.6万亩，西甜瓜2.1万亩，商品蔬菜5.4万亩，百里八角长廊新增八角林3.5万亩。水果、蔬菜良种覆盖率分别达50%和71%。林业速生丰产林建设及退耕还林工程、农村生态能源建设等工作取得新进展。以牛品改、瘦肉型猪、网箱养鱼、三黄鸡等为重点的养殖业发展加快，水产畜牧业占农业总产值的比重达40%。现代农业实验区建设成效明显，得到了自治区的肯定。成功地承办了“富民兴桂人才创业论坛——广西现代农业发展与引智引资梧州研讨会”。各核心示范园区建设取得新进展。全面完成了水利除险加固任务。抗洪救灾取得重大胜利，灾民生活得到较好安排，建成了横桥新村等一批灾民新村，恢复生产、重建家园工作取得较大成绩。

国有资产经营初见成效，国企改革取得实质性突破。为加快国有企业的改革，成立了东晖、东旭国有资产经营公司，大胆探索有效的国有资产管理方式，并取得了明显效果。共制订了14家国有企业产权改革和盘活资产的方案并予以实施，已改制的国有企业资产占全市国有企业总资产的近50%，是历年来改革力度最大的一年。利用外资改造国有企业取得突破性进展，合同利用外资额1.54亿元，基本完成了奥奇丽公司、玻璃厂、新华电池公司、印染厂、制锁厂等企业股权（产权）出让工作，实现国有企业产权多元化，促进了企业规模的扩张和资产的盘活。减掉特困企业债务4.69亿元，使一些特困企业重新获得生机。完成了康达公司的资产重组工作，更名为广西红日公司。成立了桂江电力股份有限公司，妥善处置京南电站债权债务问题。企业技术创新有了新的进步，全市工业企业技改投入2.3亿元，比上年增长 56%。规模以上工业企业新产品产值完成3.53亿元，增长41%。加强国有企业管理，委派国有企业财务总监工作取得新进展。大宗物资公开采购取得新成果，共采购物资4.4亿元，占采购物资总额的60%，节约资金1913万元。外向型工业园区建设步伐加快，首期开发360亩土地的基础设施和配套工程正在抓紧施工，引进了广东鹤山银雨灯饰公司。全市规模以上工业企业完成工业增加值16.78亿元，增长9.9%；规模以上工业企业盈亏相抵后实现利润8076万元，增长10.8%。其中国有及国有控股企业盈亏相抵后实现利润3538万元，增长106.2%，为1994 年以来的最好成绩。

重大项目前期工作扎实推进，基础设施建设步伐加快。事关梧州经济快速发展的“五大工程”，经过我市努力争取，前期工作或建设进展较好。长洲水利枢纽工程左右岸进场道路已开工建设，项目的立项已通过国家计委的审查，待国务院审批。洛湛铁路永州至岑溪段全线的定测和初步设计已经完成，可行性研究报告和初步设计评估均获通过，有望在今年上半年开工建设。桂梧高速公路桂林至梧州段前期工作正在抓紧进行，业主已基本落实，苍梧至郁南段已全线开工建设。南梧高速公路岑溪至梧州段的可研报告已获批复。河东防洪堤在遭受多次洪水袭击的情况下，工程质量和施工进度仍得到保证，主体工程已按计划完成至20米高程，为今年5月31日前完成主体工程并发挥防洪作用奠定了坚实的基础。“骑楼城”一期工程已经启动。

城市建设步伐加快。完成了梧州市城市总体规划修编工作。新兴一、二路示范街改造工程、市政广场二期工程、宝石城、联通GSM五期工程、第七期数字移动通信扩容工程已建成或投入使用。市区东南出口道路拓宽改造、新汽车客运中心、新工人文化宫、新迎宾馆、市政大厦、广电中心和城市电网改造等基础设施项目相继开工建设。新世纪大厦、金苑时代广场等大型商住楼、城市亮化等工程顺利推进。各县（市）城区面貌有了新的改观，城区街道改造、绿化力度加大，苍梧世纪广场、藤县西江大桥、藤县县城防洪堤、蒙山县东江路改造等工程已建成或顺利推进。小城镇建设也有新的进展，一批城镇基础设施项目相继竣工。

发展环境进一步改善，对外开放进一步扩大。加强投资软环境建设，出台了《梧州市改善投资软环境的意见》，聘请了一批投资软环境监督员，继续发挥“三个中心”的作用，为外商提供的各项服务进一步改善。在全区投资软环境综合评估中，我市获得第二名的好成绩。进一步提高通关速度，强化口岸协调工作，整顿港口装运秩序，确保每天有货轮出港。下大力气抓好重点产品出口，加大扶持民营企业产品出口的力度。指导和鼓励出口企业在梧州办理出口业务，帮助企业解决出口周转金困难问题，有效地减少了出口商品外流。市属外贸出口大幅度增长，首次突破1亿美元大关，增长40.7%，高出全区平均水平18个百分点。招商引资采取新措施，组建了招商专业队。全年新批外商投资项目46项，实际利用外资继续保持增长。整顿和规范市场经济秩序取得阶段性成果。非公有制经济进一步发展，全市新增私营企业312家，新增个体工商户12330户，非公有制经济已成为市场商品销售的主力军，共实现消费品零售额46亿元，占整个市场份额的82.3%。

财政改革稳步推进，金融保险事业进一步发展。去年我市克服了工业新上项目和技改投入不多，政策性减收因素等各种困难，强化税收征管，防止新欠，清缴旧欠，严厉打击偷、逃、骗、抗税等违法行为，大力组织收入，超额完成财政收入增长9%的目标任务，高出全区平均水平1.1 个百分点。支出结构进一步优化，千方百计保证了干部职工工资发放，全市没有发生新的欠发工资，市直还保证了“两项补贴”的发放。进一步推进财政管理体制改革，在巩固完善财政统发工资、政府采购、部门预算、委派国有企业财务总监等改革成果的基础上，大胆探索和制定了城区之间、市本级与城区之间的税收征管办法，规范了市区税收征管、国库分级管理的秩序，避免了互挖税源的现象，促使各城区集中精力向外招商。为支持经济发展，市本级财政投入工、农、贸产业资金2563万元，减免企业债务7161万元。金融保险事业进一步发展，年末金融机构各项存款余额129.17亿元，比年初增长9.6%；各项贷款余额82.26亿元，比年初增长5.4%；各险种投保金额416.76亿元，增长31.4%。

科技教育取得新进步，各项社会事业全面发展。进一步加快科技兴市步伐，全面实施新一轮科技创新计划，继续深化科技管理体制改革。加大对全市教育事业的投入，教学条件进一步改善，基本完成全市中小学校布局的调整，职业教育、高中教育规模扩大，西大梧州分校扩建扩招完成预期计划。教育质量不断提高，高考成绩继续位居全区前列。文化事业又有新举措，广场文化活动丰富多彩。乡村卫生服务一体化和城市社区卫生服务试点工作顺利推进，疾病控制和预防保健工作得到加强，城乡医疗卫生服务水平进一步提高。农村基层基础设施建设工作扎实开展，除自治区安排项目外，我市自筹资金和部门帮扶增加项目88 个。全市共新建、扩建和维修教育用房、卫生院、镇村办公楼项目共516个，改善了农村基层基础设施。完成了县（市、区）、镇（乡）和村委会的换届选举工作，县乡（镇）领导班子得到加强，农村基层政权进一步巩固。社区建设步伐加快。计划生育工作完成较好，资源、环境协调发展。旅游事业又有新发展。国防和民兵预备役工作进一步加强。体育、审计、统计、物价、地方志、档案、新闻出版、广播电视、人民防空等各项工作在经济建设和社会发展中作出了积极贡献，民族宗教、对台事务、外事、侨务、老龄、妇女儿童和残疾人等事业也取得了新的成绩。

社会保持稳定，安全生产工作成效明显。加强社会治安综合治理，以公安机关“阳光工程”建设为载体，提高了对社会治安的防范控制能力。深入开展“严打”整治斗争，打掉了一批社会黑恶势力。加大对“法轮功”邪教组织的打击力度，积极调处农村“三大纠纷”，确保了社会的稳定。全年做到“大事不出，中事不出，小事少出”，社会稳定工作得到了自治区的肯定。完善社会保障体系，扩大社会保险覆盖面成效显著，进一步巩固了“两个确保”，使企业离退休职工和下岗职工百分之百按时足额领到养老金或基本生活费。高度重视就业和再就业工作，千方百计为下岗职工、困难职工扩大就业机会，全年共安排了3080 名城镇失业人员再就业，城镇登记失业率控制在3.5%以内。劳动和社会保障工作获得了国家劳动和社会保障部的表彰奖励。落实城镇居民最低生活保障制度，实现了应保尽保。高度重视安全生产工作，强化责任，加强督办，狠抓落实，杜绝了重特大安全生产事故的发生。安全生产工作得到了国家安全生产监督管理局的表彰奖励。

精神文明和民主法制建设得到加强，依法行政水平不断提高。深入学习贯彻党的十六大精神，各级领导干部执政为民的思想和实践“三个代表”的自觉性进一步增强，干部群众的思想政治素质明显提高。深入开展文明创建活动，获得中国优秀旅游城市、自治区文明城市、双拥模范城、城市市容环境综合整治“南珠杯”优秀城市奖等荣誉。主动接受人大监督，定期向人大常委会报告工作，支持政协参政议政，自觉向政协通报情况，加强与各民主党派、工商联及人民团体的沟通和联系。认真办理人民代表议案、建议以及政协委员提案。结合应对入世，清理和规范了一批政府文件，不断提高依法行政水平。加大普法力度，广大干部群众的法制观念得到加强。机构改革后，公务员队伍素质进一步提高。开展万人评议机关作风活动，政府机关工作作风进一步转变。加强对重要工作和重大事项的督办力度，重视群众的来信来访工作，及时解决群众反映强烈的热点、难点问题，提高办事质量和效率。坚持不懈地抓好反腐倡廉工作，严肃查处违法违纪案件，党风廉政建设取得了新的成效。

各位代表，过去的一年，我市国民经济和社会各项事业获得了较快的发展，成绩来之不易。这是在中共梧州市委的正确领导下，在市人大及其常委会的监督和市政协的支持下，全市广大干部群众与时俱进，开拓创新，顽强拼搏，共同努力的结果，也是与驻梧人民解放军、武警部队官兵和中央、自治区有关单位以及海内外各界人士的大力支持分不开的。在此，我代表市人民政府，向为我市改革与发展作出积极贡献的广大干部群众，向驻梧人民解放军、武警、预备役官兵，向驻梧中央、自治区各单位，向所有关心支持我市改革与发展的各界朋友，致以衷心的感谢和崇高的敬意！

在肯定成绩的同时，我们也清醒地看到，我市在加快发展的过程中，还存在一些不容忽视的困难和问题。主要是：经济总量偏小，税收大户不多，财政收支矛盾比较突出；有的企业改革步伐不快，非公经济成份占国民经济比重较少，部分企业生产经营困难；投资环境有待进一步改善，招商引资发展不平衡；农民收入增长缓慢，部分群众尤其是弱势群体的生活困难，社会保障以及就业和再就业压力仍然较大；城镇化建设有待加快，城市管理水平有待提高；一些部门主动为群众和企业服务意识不够强，廉政建设有待进一步加强等。对此，我们必须高度重视，在今后工作中认真加以解决。

二、深入贯彻党的十六大精神，加快建设广西东大门步伐，努力实现梧州跨越式发展

今年是党的十六大提出全面建设小康社会奋斗目标的第一年，也是我市能否顺利完成“十五”计划的关键之年，我们要做好今年工作，就要认真贯彻落实党的十六大精神，进一步认清形势，再次解放思想，提高认识，紧紧抓住工作重点，充分发挥梧州区位优势，真抓实干，奋力拼搏，努力实现梧州跨越式发展。

要实现梧州跨越式发展，必须深刻领会全面贯彻党的十六大精神。党的十六大是我们党在新世纪新阶段召开的一次重要的历史性大会。大会报告科学总结了改革开放以来特别是十三届四中全会以来党领导人民建设中国特色社会主义的辉煌成就和基本经验，进一步阐明了贯彻“三个代表”重要思想的根本要求，提出了全面建设小康社会的奋斗目标，并制定了推进经济、政治、文化等各方面工作的方针政策。当前，我们的中心任务就是要认真学习、深刻领会、准确把握、全面贯彻党的十六大精神，把全市干部群众的思想、行动统一到党的十六大精神上来。“三个代表”重要思想是党的十六大报告的灵魂，我们必须充分认识和深刻理解“三个代表”重要思想的科学内涵和重要意义，使“三个代表”重要思想真正成为我们今后工作的指导思想和行动指南，用“三个代表”重要思想统揽全市各项工作，坚持把发展作为执政兴国的第一要务，坚持以经济建设为中心，真正做到一心一意谋发展，聚精会神搞建设，加快全市人民奔小康的步伐。学习贯彻党的十六大精神，必须与时俱进，在开拓创新上下功夫。能否做到用新的发展理念和工作方法去思考梧州的经济发展，事关梧州在新一轮大发展中的兴衰成败。学习贯彻党的十六大精神，就要结合梧州的实际，以“三个代表”重要思想为指导，以加快建设小康社会为目标，以正在做的工作为基础，围绕自治区“富民兴桂新跨越”的战略部署，抓住西部大开发的机遇，发挥紧靠粤港澳的优势，抓住基础设施、产业调整和对外开放的重点，加快工业化、城镇化和农村经济产业化进程，全面完成今年各项工作任务，努力建好广西东大门，实现梧州跨越式发展，以实际行动贯彻落实党的十六大精神。

要实现梧州跨越式发展，必须解放思想，与时俱进。与时俱进，开拓创新是贯彻“三个代表”重要思想，加快梧州发展的关键所在。要坚决冲破一切妨碍发展的思想观念，克服凭老经验、老办法做事的保守思想；克服从本本出发，以书为上，不重视非公经济发展、不结合梧州实际的教条主义思想；克服不顾梧州发展大局和全市人民根本利益的部门利益至上的思想；克服易于满足，不思进取，不致力于大规划、大动作的慢慢干思想；克服办事拖拉推诿，不负责任的官僚主义思想。用创新的思维、创新的举措去谋划工作、开展工作，努力做到在发展中探索新思路，在改革中实现新突破，在开放中开创新局面，在各项工作中推行新举措。使全市上下树立只争朝夕、力争上游的精神风貌，形成大胆探索、勇于创新、奋发有为的良好氛围。

要实现梧州跨越式发展，必须充分发挥区位优势。根据我市的实际情况，要大力发展生产力，实现我市经济快速发展，必须充分发挥区位优势，下大力气抓好招商引资工作。我市毗邻粤港澳，无论是在区位上，还是在经济纽带上，以及血缘、语言、文化的联系上，都有着独特的优势。我们的希望在区位，出路在招商。自治区提出的梧州要发挥区位优势、建设好东大门的决策是十分正确的。但当前我市一些部门对开门搞改革、利用外资搞建设认识不足，劲头也不够大。强调利用外资困难的多，想办法引资的少，知道区位优势的人多，真正发挥优势的人少，区位优势未能充分发挥。优势不是优惠，只有通过挖掘，通过艰苦的努力才能变成现实。因此，我们必须认真研究和发挥我市毗邻粤港澳背靠大西南的区位优势，在降低门槛，提高服务质量，改善招商环境，提高招商效率上下功夫，全力抓好招商引资工作，促进我市经济和社会的快速发展。

要实现梧州跨越式发展，必须尽快改善以交通为重点的硬环境。可以说，梧州过去的辉煌得益于交通，现在发展相对滞后的症结也在交通。因此，要实现梧州跨越式发展，必须尽快改善以交通为重点的硬环境。这是梧州能否抓住机遇，赶上全区中上水平的关键，全市广大干部群众特别是领导干部对此必须要有充分的认识。当前，要突出抓好三个方面的工作：一是要主动支持配合建设好三条路（一条铁路、两条高速公路），从根本上解决梧州交通滞后问题，使梧州成为广西东通道的核心城市，成为西部与粤港澳物流的枢纽。二是千方百计开工建设长洲水利枢纽工程，壮大梧州经济。三是要下大力气，搞大动作，抓好以城市防洪工程为重点的城市基础设施建设，提高梧州的城市品位，为经济发展创造一个优良的环境。只有交通方便了，电力充足了，城市变美了，环境改善了，梧州才有可能成为现代物流的中心、外商投资的热点，才更具备快速发展的条件。

要实现梧州跨越式发展，各级领导干部必须带头实干。“政治路线确定之后，干部就是决定的因素”。作为领导干部，首先要树立全心全意为人民服务的思想，坚持艰苦奋斗的优良作风，把代表人民的根本利益作为我们一切工作的归宿和出发点。要一心扑在工作上，团结和带领全市人民同心协力，艰苦奋斗，不断创造新业绩，开拓新局面。其次，要切实转变作风，带头实干。领导干部既要抓全局、抓大事、抓重点，又要抓关键的“小事”；既要作决策、发号召，又要身体力行，做好表率；既要当好领导，又要深入基层调查研究，帮助群众办好事办实事，遇到急、难、险、重的情况，领导干部要靠前指挥。绝不能片面地强调抓宏观，而忽视抓落实，使工作流于形式，影响各项任务的完成和跨越式发展的实现。再次，要注重抓实效。各级领导干部要力戒虚浮，真抓实干，弘扬艰苦朴素、勤俭办一切事业的精神。既要解放思想，开拓创新，又要实事求是，量力而行；既要抓示范，更要讲实效。不弄虚作假，不搞数字游戏，不搞“形象工程”，要多为群众着想，多给群众实惠。

三、2003年的主要工作任务

2003年是全面贯彻落实党的十六大精神的第一年，也是我市实施“建设东大门，实现新跨越”战略，全面建设小康社会的重要一年。我们要坚定不移地贯彻落实自治区党委关于“富民兴桂新跨越”的总体部署，按照自治区经济工作会议的要求，结合我市建设小康社会重点是工业，难点在农村，出路在招商的特点，与时俱进、开拓创新，提出新思路，采取新举措，努力做好今年的工作，不断开创我市改革开放和现代化建设的新局面。

我们一定要高举邓小平理论和“三个代表”重要思想的伟大旗帜，认真贯彻党的十六大和自治区党委八届三次全会、自治区十届人大一次会议、市委十届九次全会精神，坚持以经济建设为中心，以加快发展为第一要务，进一步深化改革，全面提高对外开放水平，加快经济结构调整，加强农业基础地位，推进工业化和城镇化，加快基础设施建设，促进国民经济持续更快发展。正确处理改革稳定关系，做好就业和再就业工作，完善社会保障体系，提高城乡居民生活水平，保持社会稳定。据此，2003年全市经济与社会发展的主要预期目标初步安排为：

——国内生产总值增长10%。

——财政收入增长10%。

——全社会固定资产投资增长15%。

——市属外贸出口增长10%。

——实际利用外资增长10%。

——全社会消费品零售总额增长9%。

——城镇居民人均可支配收入增长8%。

——城镇登记失业率控制在4.5%以内。

——农民人均纯收入增长6%。

——人口自然增长率控制在10‰以内。

为实现上述目标，要着重抓好以下十方面的工作：

（一）开门改革，借力发展，把工业企业做大做强

把加快工业发展，做大做强工业，放在梧州经济工作的首位。按照“开门改革、借力发展”的指导思想，明确职责，落实任务，积极引进外资、民营资本改组改造一批国有企业。充分发挥东晖、东旭两家国有资产经营公司的作用，提高其投融资能力，使其成为改制和结构调整的主体。继续完善奥奇丽公司、新华电池公司、玻璃厂等企业的改制工作。抓好中恒集团和制药集团、光学仪器厂、蓄电池厂等一批国有企业的资产重组工作。力争基本完成市区国有企业的改组改制任务，做大做强现有优势企业。处置一批不良资产，力争梧锅集团公司、梧起集团公司等特困企业的资产重组工作有新的突破。规范公司制改革，加强现代企业制度建设，真正转换经营机制。支持梧州中恒、广西红日两家上市公司增强再融资能力，引导其募集资本的投向，为梧州经济发展多作贡献。继续深化集体企业改革，支持和帮助多种形式的集体经济发展。重视并做好重组改制企业职工的思想政治工作，确保企业改革工作顺利进行。

重视技术改造工作，鼓励并支持优势产业和骨干企业进行技术改造，不断增强发展后劲。全力抓好自治区重点扶持的新华电池公司6500万只碱性电池、制药公司GMP改造、三威林产公司扩建工程、苍梧县120万吨水泥厂、蛋白肠衣厂等五个项目，加快一批产业项目的前期工作，力促优势企业上规模、上水平。重点抓好嘉进药业公司技术改造、桂西玉兰制药有限公司中成药扩产、三鹤药业公司五淋化石丸扩产等技改项目，以及新上马的郊区永达金属制品厂、苍梧金属制品厂、苍梧不锈钢厂、藤县铝箔厂等项目，使之成为我市今年新的经济增长点。大力推进外向型工业园区建设，结合我市产业优势和资源条件，积极引进项目和资金，力争年内有一批企业到园区落户。

加强企业管理。坚持国有企业财务总监委派制度，继续推行国有及国有控股企业大宗原材料公开招标采购，加强对重点企业尤其是骨干企业经济运行情况的监控，协调解决经济运行中出现的问题。指导帮助亏损大户制定扭亏增盈的措施，努力降低亏损面和亏损额。建立和完善企业内部质量管理体系，鼓励和支持企业通过质量体系认证和产品认证，进一步提高我市产品质量的整体水平。走新型工业化道路，积极推进企业信息化建设，年内要收到初步的成效。今年，全市工业增加值计划增长10%，其中规模以上工业增加值增长9%，全市规模以上工业企业盈亏相抵后实现利润增长9%。

（二）加快农业和农村经济结构调整，增加农民收入。

要把解决好农业、农村和农民问题作为今年工作的重中之重。进一步解决好“三农”问题，加快农业和农村经济发展。

加大农业结构调整力度。农业结构调整要以增加农民收入和全面建设小康社会为目标，以市场为导向，继续抓好“六大产业”开发和“六带一廊”建设，启动百里花卉长廊、百万亩速生丰产林、百万亩玉桂项目，大力发展特色经济。要以抓好现代农业实验区建设和示范推广，通过招商引资和扶持办好各种龙头企业，扶持种养专业村、专业户，抓好农村流通队伍和流通网络建设等为主要手段来拉动农村产业结构调整，推进农村产业化经营。今年优质稻种植比例要提高到75%以上，“万元田”达到30万亩。新种速生丰产林30万亩，玉桂6万亩，蚕桑3万亩，荔枝、龙眼、沙田柚、椪柑等名优水果6万亩，实现建设10万亩百里八角长廊目标。三黄鸡、瘦肉型猪等特色产品养殖，牛猪品种改良，江河网箱养鱼等要得到进一步发展。

加强农业和农村基础设施建设，加大对农业重点项目的支持力度。搞好冬春水利大会战，重点抓好城乡防洪工程、病险水库除险加固、水毁工程修复和农村饮水解困工程等基础设施建设。积极做好退耕还林工作，改善农村生产生活条件和生态环境，增强农业防灾抗灾能力。

适应新形势下对“菜篮子”工程的要求，加快农产品质量体系建设，推进农产品标准化生产，建立农产品市场质量安全准入制度和检验检测体系，禁止农药残留超标的蔬菜和未经检疫检验的畜禽产品入市销售，提高我市农产品的信誉度。

坚持党在农村的基本政策，推进农村土地依法流转和农村税费改革，减轻农民负担，大胆探索对农业和农民实行补贴的各种有效办法，保护农民利益，促进农民增收。

（三）全力推进以"五大工程"为重点的建设项目，努力改善经济建设硬环境

抓住国家继续实施积极的财政政策和加大西部地区投入的有利时机，把握国家投资方向，努力争取国家的国债资金和专项资金的支持，改善我市的基础设施。

继续做好长洲水利枢纽项目的全程跟踪落实工作，争取尽快拿到立项批文。同时，加快推进左、右岸进场道路施工和移民点建设等各项工作，为年内主体工程开工做好充分准备。河东防洪堤建设要在确保工程质量的同时，精心安排和组织好施工，千方百计加快施工进度，确保在今年5月31日前完成主体工程，并在汛期发挥防洪作用。继续配合铁道部门做好洛湛铁路项目前期工作，积极开展梧州段征地拆迁的调查摸底等工作，为项目早日开工做好准备，同时，积极争取湖南永州至岑溪段的开工仪式在我市举行。全力推进高速公路项目建设，加快桂梧高速公路苍梧至郁南段施工进度，积极跟踪落实桂梧高速公路梧州段、南梧高速公路岑溪至苍梧段的前期工作，力争今年开工建设。认真做好"骑楼城"工程项目的规划设计和招商引资工作，争取上半年完成部分街道的改造。

此外，还要开工建设潭东至太平一级公路、自治区桐林监狱搬迁等一批项目。做好潭东至新地、岑溪至罗定一级公路等项目的前期工作。开展乡村公路大会战，逐步解决乡村道路差的问题。

（四）突出区位优势，实现梧州对外开放有显著进步

梧州的优势是区位，梧州的出路在招商。我们要充分发挥东晖、东旭两家国有资产经营公司的招商主体作用，以国有企业和工业园区为招商载体，推出一批新的国企改制项目进行招商，引进有实力的大公司、大财团和利用社会民间资金，改组改造我市的国有及国有控股企业，努力使国企招商有新突破。要充实招商专业队力量，提高工作人员的素质，落实责任制，使其成为我市招商引资的主力军。要充分发挥社会各方面的积极性，共同为招商出力。要采取走出去，找上门，交朋友，动之情，给其惠的办法，动员外商到梧州投资办企业，收购重组梧州国有企业，提高招商的成功率。

努力保持进出口贸易的稳定增长。要在巩固发展西非、东盟等市场的基础上，主动研究并组织企业走出去开拓南非、欧洲和美国、俄罗斯市场。充分利用入世后部分商品放开配额限制的有利时机，继续抓好松香、电池、宝石、打火机、纺织品、电子等重点产品的出口。积极扶持企业开展加工贸易，促使加工贸易有较快增长。进一步扩大出口队伍，大力扶持民营企业、集体企业申报进出口经营权，并指导和帮助这些企业解决出口遇到的困难和问题。采取切实有效的措施，提高通关速度，大力整顿港口秩序，规范航运管理，解决好出口商品外流问题。

努力改善投资环境。进一步落实《关于进一步加强投资软环境建设的意见》，强化亲商、安商、富商的观念，想方设法降低地价和各种收费，适当控制和调低电价、水价，下大力气降低招商门槛。继续发挥“三个中心”和投资软环境监督员作用，建立服务优质、管理高效的机制，实行外商因投资环境问题外迁的部门领导责任追究制，建立相关部门主动为外商服务的检查汇报制度，促使政府部门转变观念，变被动服务为主动服务，不断提高服务质量和水平。

（五）加强城市规划、建设和管理，提高经营城市水平

加强市政建设的领导，成立市政建设重大项目办公室，在市政府的领导下，直接协调市政重大项目的建设和相关部门的工作。

强化规划在城市建设中的指导作用。按照城市总体规划和城市分区布局，完成红岭片修建性详规和长洲岛的控制性详规工作，为城市的建设做好基础性工作。

高度重视并积极做好市区行政区划调整工作。要提高认识，切实加强领导，把市区行政区划调整工作作为政府今年的一件大事来抓紧抓好，确保此项工作的顺利完成，努力把好事办好。要充分利用这一契机，做好提高城市品位、推动城区经济乃至全市经济发展、改善城区居民生活这三篇文章，使梧州市整体水平上一个新台阶。

加快市区东南出口道路拓宽改造工程、市第一污水处理厂、市供水管网改造、汽车客运中心、西大分校二期扩建、新迎宾馆、市政大厦、广电中心、新工人文化宫、河西路网改造、城市电网改造等在建城市重点项目建设，确保按时完成任务。

积极筹措资金，努力争取国债支持、银行贷款，创造条件新上一批项目。争取开工建设新兴三路延长线、红岭私宅区、万秀区地质灾害居民区改造、城市生活垃圾综合处理、殡仪馆搬迁、西堤路拓宽、桂江一桥路面维修、中山路民安广场等一批新项目。加快梧州新港、西江三桥、三龙滨江大道、火车站站前大道和火车站站前广场、城市排水系统改造、三龙和长洲防洪堤、南岸开发等一批城市基础设施项目的前期工作。

努力推进城镇化进程。落实《城市人口准入暂行办法》，降低入城“门槛”，增加城市人口。积极探索经营城市的新路子，通过资本运营，盘活用好土地等城市资产和资源，加快城市基础设施建设。抓好重点城镇发展，建设一批小城镇基础设施项目，加快城镇化步伐。

加强城市管理。理顺和完善城市管理体制，建立城市管理统一执法机构，调整、充实、提高执法队伍的素质，强化城市管理工作。继续推行“门前三包”等行之有效的办法。进一步完善环卫、绿化队伍工作责任制，推行竞争上岗，实行按卫生、绿化优劣给酬。加强城市道路交通管理，着力改变城市“脏、乱、差”的落后面貌，努力建设环境优美管理有序的城市。

（六）加强财税金融工作，进一步加大财政管理体制改革的力度

强化财源建设。市财政今年要拿出7381万元支持经济发展，主要是增加对工业、农业、科教、城市基础设施等方面的投入，增强发展后劲，确保财政收入稳定增长。

全力组织财政收入，努力增收节支。继续推行财税目标责任制，坚持依法治税，加强征管，做到应收尽收，严厉打击偷、逃、骗、抗税的违法行为，确保财政收入及时、足额、均衡入库。要贯彻"一要吃饭、二要建设"的方针，调整优化支出结构，集中资金保工资、保重点、保稳定。

继续推进财政管理制度改革。巩固和扩大政府采购、统发工资、部门预算、财务总监的改革成果，积极推进市政府集中核算支付制度改革工作，今年要在39个单位开展试点工作基础上，进一步扩大覆盖面，实施单位要取消帐户和会计，严禁设立小金库，严格按政府的审批制度核报财务，接受有关部门的财务监督管理。加强行政事业单位国有资产经营和管理。认真抓好行政事业单位国有资产的购置、调剂、使用的管理与监督，盘活闲置资产1000万元以上，增加财政收入。

加强金融监管，维护金融稳定。规范社会信用秩序，努力培育良好的信用环境，发展储蓄信贷业务，支持保险部门发展保险事业。积极争取金融部门支持重点企业和基础设施建设。继续抓好"两会一部一借"和城市信用社的清理整顿工作，防止和化解金融风险。

（七）加快服务业发展，壮大非公有制经济

加快旅游业发展。加大白云山、龙母太庙、太平狮山、飞龙湖旅游区（爽岛）、李济深故居、太平天国封王建制遗址、“骑楼城”等旅游资源的开发力度，塑造特色旅游精品。充分利用"中国优秀旅游城市"这个品牌，积极开展形式多样的旅游促销活动，提高接待能力和服务水平，争取旅游人数有较大的增加。

促进商贸大流通。建设、培育两广市场、宝石市场、建材市场、太阳广场、国泰广场，以及县乡农副产品市场，努力办出特色，形成规模，带动经济发展。以"骑楼城" 和河东防洪堤建设为契机，加快特色街、步行街、购物中心等建设，促进河东商贸中心的繁荣。配合洛湛铁路和高速公路建设，抓好我市现代物流业特别是服务业的发展。继续整顿和规范市场经济秩序，严厉打击制售假冒伪劣商品的行为。

大力发展非公有制经济。进一步完善和落实关于鼓励非公有制经济发展的各项优惠政策，降低市场准入"门槛"，努力为非公有制经济发展创造公平、宽松的竞争环境。要打破分所有制扶持的旧观念，只要符合国家产业政策，只要有利于梧州经济的发展，不管是什么所有制，市政府将一视同仁，给与政策上、经济上的扶持。要支持和帮助非公有制企业加快技术改造和技术创新，促其向科技型、规模化和创名牌方向发展。

（八）不断完善社会保障制度，积极扩大社会就业，努力提高人民生活水平，维护社会稳定

加快社会保障体系建设，提高社会保障能力。各级政府要把“两个确保”和最低生活保障工作摆在突出位置抓紧抓好，巩固和完善三条“保障线”。进一步抓好养老保险的扩面和基金征缴工作，确保企业离退休人员基本养老金按时足额发放。在加快推进国有企业下岗职工基本生活保障向失业保险并轨的同时，对仍在再就业服务中心的下岗职工，继续按照“三三制”原则安排资金，确保下岗职工基本生活费按时足额发放。完善城市居民最低生活保障制度，确保实现应保尽保。依法扩大失业保险、医疗保险的社会覆盖面，提高保险费的收缴率。建立社区劳动保障机构，做好企业离退休人员社会化管理工作。继续实施送温暖工程，切实帮助困难群众和弱势群体解决生产生活中的实际困难。加大对灾区生产自救、重建家园的扶持力度，确保灾民的基本生活。进一步加强新时期的扶贫开发工作，大力实施科技扶贫和生态扶贫，扩大扶贫成果。

广开就业渠道，扩大社会就业。加快国有企业改制和做大做强的步伐，继续抓好乡镇企业，积极发展多种所有制经济，特别是个体私营、股份合作等非公有制经济发展，鼓励和支持劳动密集型产业的发展，增加就业岗位。加快劳动力市场建设，通过市场配置大力组织劳务输出，减轻就业压力。继续落实再就业优惠政策，实施再就业工程，抓好下岗失业人员职业技能培训，加强职工的就业观念教育，鼓励倡导自谋职业、自主创业，提高再就业率。

努力维护社会稳定。继续抓好“阳光工程”建设，提高政法队伍的整体素质和办案水平，严格公正执法。以“打黑除恶”为重点，深入开展“严打”整治斗争，坚决铲除社会黑恶势力，严厉打击各种刑事犯罪和“法轮功”邪教组织。坚持社会治安综合治理，积极开展社会治安专项整治行动，强化综合治理基层基础工作。加大对农村“三大纠纷”的调处力度，认真排查各种矛盾纠纷，有效地化解矛盾，预防和妥善处理群体性事件。

切实抓好安全生产，强化各级各部门安全生产责任制，突出重点，加大对重大隐患的跟踪和整改力度，防止重大安全事故的发生。

（九）加强精神文明建设，坚持实施科教兴市和可持续发展战略，提高社会各项事业发展水平

大力加强社会主义精神文明建设。认真抓好以诚信建设为重点的公民道德建设，进一步加大《公民道德建设实施纲要》宣传力度，加强社会公德、职业道德、家庭美德教育，提高社会公共道德水平。深化文明创建活动，大力创建文明城市、文明单位、文明村镇、文明社区。

加大科教兴市工作力度。加快推进农村信息化体系与示范应用项目建设，建立农村科技信息四级网络，大力推广农业新品种、新技术。加大科技创新力度，依靠科技改造传统产业，提高新产品的产值率。各级政府要切实把教育放在优先发展的地位，加大对教育的投入，积极鼓励和引导多种形式的社会办学，全面推进素质教育，提高教育质量。完善市区中小学校布局，建设第15中学、新兴二路小学。完成农村中小学校10万平方米危房改造，争取用2至3年时间基本解决全市中小学危房问题。启动“市区校校通”工程。抓好职业教育，加快示范性高中建设步伐。抓好西大梧州分校扩招和二期扩建工程，确保如期完成建设任务。重视抓好引进人才工作，充分发挥现有人才的作用。按照自治区的部署，积极推进以聘用制度和岗位管理为重点的事业单位人事制度改革。

积极发展文化、卫生、体育事业。开展经常性的文化下乡、文化进社区和广场文化等活动，抓好乡镇文化站建设，丰富群众文化生活。筹拍电视连续剧《李济深》，扩大梧州的影响。努力发展卫生事业。建立和完善农村卫生管理体制和农村卫生服务体系，开展新型农村合作医疗试点，改善农村卫生环境，解决农民看病难的问题。改革市区卫生医疗机构，合理配置和利用卫生资源，避免不当竞争。加强城市社区卫生服务工作，强化政府对公共卫生的管理职能。大力开展群众性体育活动，努力提高全市人民身体素质，不断向上输送体育人才。

实施可持续发展战略，促进人口、资源、环境与经济社会协调发展。加强计划生育工作，促进优生优育，巩固低生育水平，提高人口素质。依法加强土地、矿产和水资源管理，合理开发利用资源。抓好国土资源的规划和管理，加强基本农田保护，统筹安排各类建设用地，确保全市耕地总量动态平衡。重视生态环境保护，加大治污力度，提高城乡环境质量。关心和支持驻梧部队、民兵预备役建设，继续做好双拥工作，进一步密切军政军民关系。审计、统计、新闻出版、人民防空、对台事务、外事、侨务、地方志以及老龄、妇女、儿童、残疾人等事业也要有新的发展。

（十）切实加强政府自身建设，塑造政府新形象

注重务实高效。各级政府和全体公务员要牢记全面建设小康社会的历史重任，增强使命感和责任感，自觉为党和人民的事业奋斗。继续推行万人评议机关作风活动，切实转变机关作风，提高行政效能。各级政府各部门要把主要精力集中到抓工作的落实上来，减少“文山会海”，减少事务性活动。完善岗位目标责任制，强化工作督查制度和责任追究制度，确保各项任务的完成和政令的畅通。各部门要从全市经济大局出发，坚决反对部门利益至上和互相扯皮的不良作风。

严格依法行政。各级政府要自觉接受人民代表大会及其常委会的监督，完善工作报告制度，认真执行和落实有关决议决定。积极支持人民政协履行“政治协商、民主监督、参政议政”的职能，认真办理人大代表议案、建议、批评、意见和政协委员提案，提高办理质量。加快基层民主建设，推进政务公开。规范政府行为，出台政策要有依据，行政行为要依法进行，努力提高依法行政水平。

为民多办实事。各级领导要牢固树立执政为民的思想，树立正确的权力观，增强公仆意识，努力把代表最广大人民群众根本利益的要求体现在行动上、落实在工作中，增进政府与人民群众感情。坚持和完善领导干部定点联系乡镇、村、企业等制度，多为群众办实事好事。重视调查研究和群众来信来访工作，切实解决群众普遍关注的热点问题。

保持清正廉洁。切实加强政府廉政建设，做到勤政为民，廉洁奉公。加强对政府采购、工程项目招投标、土地使用权出让、产权交易等行为的规范管理和依法监督，确保其公平、公正、公开、高效进行，从源头上预防和治理腐败。坚决查处以权谋私、贪污腐化、侵害群众利益等违法违纪行为。

各位代表，党的十六大为我们描绘了全面建设小康社会的宏伟蓝图，指明了前进的方向。当前我市正处在发展的关键时期，面临着良好的发展机遇，基本具备了加快发展的条件。在新的一年里，让我们紧密团结在以胡锦涛同志为总书记的党中央周围，高举邓小平理论伟大旗帜，努力实践“三个代表”重要思想，全面贯彻落实党的十六大精神，牢记“两个务必”的要求，在中共梧州市委的领导下，团结和依靠全市人民，抢抓机遇，与时俱进，开拓进取，为建设东大门、实现梧州新跨越而努力奋斗！
